# A Report on the Progress of Opthalmology and Rhinology*Read before the Medical Association of Georgia, at Brunswick, Georgia, April 18th, 1890.

**Published:** 1890-05

**Authors:** R. O. Cotter

**Affiliations:** Macon, Ga.


### A REPORT ON THE PROGRESS OF OPTHALMOLOGY AND RHINOLOGY.*

BY R. O. COTTER, M. D., MACON, GA.

I am well aware that an attempt to present anything like a *resume* of the progress made in any special line of practice would be out of place, and tiresome to an audience composed largely of general practitioners; hence, I shall endeavor to prerent only some of the more important and lately known measures in the treatment of diseases of the eye and nose.

### EXTRACTION OF CATARACT WITHOUT MAKING THE IRIDECTOMY.

This (so-called) new operation, which is, and has been quite the fashion with some surgeons in this country for a year or so, is simply the revival of an old operation which was practiced in France some thirty years ago. Its enthusiasts claim that, among other things it offers and secures by the circular and movable pupil which it leaves, as a rule, better vision than is obtained by the usual operation *with* iridectomy. I refer to

*Read before the Medical Association of Georgia, at Brunswick, Georgia, April 18th, 1890.

Von Graefe's linear extraction, which has given, for 25 or 30 years, such great satisfaction. I think this claim remains yet to be proven. Recently I reported the case of a patient who, from an injury received a year ago, which tore away nearly all of the iris of one eye, leaving only a narrow strip (hardly one-fourth of it) yet, with this narrow strip of iris, he has perfect vision—reads the finest test type. I first saw this new operation made last summer in New York, at the Manhattan Eye and Ear Hospital. I liked the operation, for, when successful, it seems to be really the ideal operation, and it is perfectly natural that most eye surgeons should desire to make it. But it is admitted, even by its advocates, to be a very difficult one. I go further and say, it is an exceedingly *hazardous* one. I am very sorry too that it is so, because I am confident that, very many eyes will be lost by it before it shall have been abandoned, as I think it will be before very long by conservative surgeons. What I now have to say, is an expression of my own opinion, as well as it can be formed from having made the operation nine times myself, and from some experience of others whom I know. Of my nine cases, eight were successful, and one was a complete failure. These eight successful ones now enjoy good vision, but during the progress of the operation of three of these, complications arose, which I almost feared would ruin the success of the operations. These mishaps I have seen occur to the most skilled and careful operators, and they can not certainly be anticipated or prevented. Such mishaps are extremely rare with me when I employ the Graefe's extraction.

In the first place, the circum-corneal incision has to be very extensive—fully two-fifths of the circumference. This augments the danger of non-union and suppuration. Then, as we wish to keep the pupil contracted, we cannot use that great sedative, Atropine. Indeed, on the other hand, it is recommended that we drop a solution of eserine in the eye after extraction. As we know eserine is a drug which is capable of doing mischief by its irritating properties, I regard this as unfortunate. Loss of vitreous, while it may not be one of the commonest mishaps that may occur, is the worst accident that is liable to occur, and it is surely more liable to occur than it is in the Graefe's extraction. This is because of the extra large circum-corneal cut, and that the lens comes out with more of a gush than in the usual operation. During all of my former experience in operating I was so fortunate as to have never had any patient suffer a serious loss of vitreous. In my third case of the new extraction, however, after the lens had been delivered, the patient, a silly, uncontrollable old woman, foolishly snapped her lids together as strongly as she possibly could—though there was no pain whatever. A large amount of vitreous gushed out and the eye was lost. By-the-way, I notice recently that loss of vitreous after the operation had been completed, was reported as having occurred among the patients at the Manhattan Hospital, presumably in somewhat the same manner as in my case. So far as my experience goes, I have had no iritis as the result in any of my cases, and I should think this is in favor of the new operation, because with an uncut iris it seems reasonable that there should be less danger of iritis. However, as I have for the past five years adopted the rational plan of "letting the eye alone" after the operation (I mean, not disturbing the eye for two or three days if possible), I have a minimum amount of iritis to deal with any way. Another point I will mention in favor of the new operation is this—although I had about decided about six weeks ago that I would, as a rule, abandon the operation, since then I have been somewhat forced to make it in two more cases. These were cases when the patients were exceedingly scary and nervous; they constantly jerked and rolled their eyes in all directions. Now, in such cases there is often a very provoking and dangerous jerk of the eye just as we grasp the iris to make the iridectomy, in the Graefe's operation. Frequently this causes very troublesome hemorrhage, and sometimes does worse than this. So in these cases I found it necessary to deliver the lens without making the iridectomy. I suppose the most frequent accident that occurs in this operation is prolapse of the iris. Indeed, it is to be expected, and when primary, is, so far as I have seen, usually reducible, but when secondary, is apt to prove an annoying and tedious matter.

Subsequent incarceration of the iris in the wound, is also said to be a tolerably frequent occurrence. If I am positive of anything in regard to what I have seen in cataract extractions (and I will say they were not my own cases) it is that, I have known more unsuccessful results follow from the meddling, or too frequent dressings of eyes than from all other causes combined. If I can possibly avoid it, I do not remove the dressing for two or three days, nor do I ever open the lids until the fourth or fifth day, if there is no swelling of the lids and other symptoms. Now, suppose you have made the extraction by the new method and all goes well for two or three days (as was my experience in one case, and it was one of the most successful, and as smooth an operation as I ever saw) but about the second or third day the compress or the bandage should get slightly awry, or the patient move his lids (and they will do so) the aqueous comes out with a gush, and the iris becomes prolapsed. This is, in my opinion, *very* liable to occur. Indeed, this was the tendency in the majority of my cases. Fortunately I overcame the trouble in all of them; but one of them was a very tedious affair, and kept the patient from going home for at least four weeks, though she now has good vision. During one of my operations the iris fell forward upon my knife, and for sometime it was quite troublesome. This is a very common hindrance to the operation I should say, and I should think, to any one but a skillful operator, it would be liable to cause serious mishaps. It seems liable to cause the line of incision to be deflected, or to gash the iris. I will also say, I find it best and safest in making this operation to remove the speculum as soon as the circum-corneal incision is made. I may appear to be out of fashion in having to condemn this operation, but I believe the progress of opthalmic surgery will yet sustain me in my opinion. I believe that if the records of failures were brought to light, it would show that many eyes are being sacrificed by it, which could have been restored to vision by Von Graefe's method.

In spite of the fact that so much has been written regarding the improper use of cocaine in eye troubles, I am sorry to have to repeat that, I too frequently find this most valuable boon to the opthalmic surgeon being used in a very improper manner by some good physicians. They persist in using it as a sedative, or as a collyrium. Let me most earnestly protest against such a use of cocaine. The continued use of it, as in eye washes, is positively harmful. It denudes the cornea of its epithelium, and when used, as it often is, in ulcers of the cornea, it does great harm. As for the use of Atropine—the most valuable of all eye medicines when properly used—yet, its indiscriminate use often does irreparable injury to the eye. My apology for repeating this well-known truth is that, within the past twelve months I have known of four or five instances where general practitioners (and men of undoubted ability, too,) had used atropine in making examinations, or improperly in the treatment of eyes of patients who had glancoma, some incipient, and some established cases. In all these cases serious harm was done.

Since reading my paper upon the correction of errors of refraction, at our meeting of last year, I am confident that I hardly made it strong enough. I am more than ever convinced that, more and more cases of conjunctivitis, and frequently resultant granulated lids, have their origin solely in this cause. I am more than ever convinced that, many hypermetropes are made near-sighted by being allowed to continue close use of their eyes with uncorrected errors of refraction. I then said that I was not ready to give my testimony as to the comparative merits of atropine, which paralyzes the accommodation for several days, and homatropine, whose effects last only about twenty-four to thirty-six hours, when used in refractive work. After a sixteen months trial of homatropine, I am very sorry to have to say it can not always be depended upon, I have used it in all sorts of ways, and as thoroughly as I have ever heard its use advised. Dropping a six, or a ten-grain-to-the-ounce-solution of it in the eye every ten minutes for an hour, and know that it will frequently fail, especially when used with young patients, most particularly where there is spasm of the ciliary muscle, and the patient simulates near-sightedness. Here atropine should be used. In adults whose time is more valuable, and also as there is greater possibility of incipient glancoma, I generally use homatropia. It does not keep the pupil so long dilated. We should bear in mind that many eye diseases, neuralgia, conjunctivitis, stricture of the nasal duct, etc., are caused and aggravated by nasal catarrh in its various forms. If we overlook this, now well-established fact, we will fail to relieve many perfectly curable eye troubles.

This brings us to the subject of RHINITIS, by which is understood, a catarrhal inflammation (if I may use the term inflammation) of the mucous membrane of the nostril and continuous passages. In this section, unless it is from syphilis, we see comparatively little of ozena, so I will not take up time with it.

(Te bc continued.)

